# Using DNA Methylation Patterns to Infer Tumor Ancestry

**DOI:** 10.1371/journal.pone.0012002

**Published:** 2010-08-09

**Authors:** You Jin Hong, Paul Marjoram, Darryl Shibata, Kimberly D. Siegmund

**Affiliations:** 1 Department of Preventive Medicine, Keck School of Medicine, University of Southern California, Los Angeles, California, United States of America; 2 Department of Pathology, Keck School of Medicine, University of Southern California, Los Angeles, California, United States of America; University of Georgia, United States of America

## Abstract

**Background:**

Exactly how human tumors grow is uncertain because serial observations are impractical. One approach to reconstruct the histories of individual human cancers is to analyze the current genomic variation between its cells. The greater the variations, on average, the greater the time since the last clonal evolution cycle (“a molecular clock hypothesis”). Here we analyze passenger DNA methylation patterns from opposite sides of 12 primary human colorectal cancers (CRCs) to evaluate whether the variation (pairwise distances between epialleles) is consistent with a single clonal expansion after transformation.

**Methodology/Principal Findings:**

Data from 12 primary CRCs are compared to epigenomic data simulated under a single clonal expansion for a variety of possible growth scenarios. We find that for many different growth rates, a single clonal expansion can explain the population variation in 11 out of 12 CRCs. In eight CRCs, the cells from different glands are all equally distantly related, and cells sampled from the same tumor half appear no more closely related than cells sampled from opposite tumor halves. In these tumors, growth appears consistent with a single “symmetric” clonal expansion. In three CRCs, the variation in epigenetic distances was different between sides, but this asymmetry could be explained by a single clonal expansion with one region of a tumor having undergone more cell division than the other. The variation in one CRC was complex and inconsistent with a simple single clonal expansion.

**Conclusions:**

Rather than a series of clonal expansion after transformation, these results suggest that the epigenetic variation of present-day cancer cells in primary CRCs can almost always be explained by a single clonal expansion.

## Introduction

**Figure 1:**
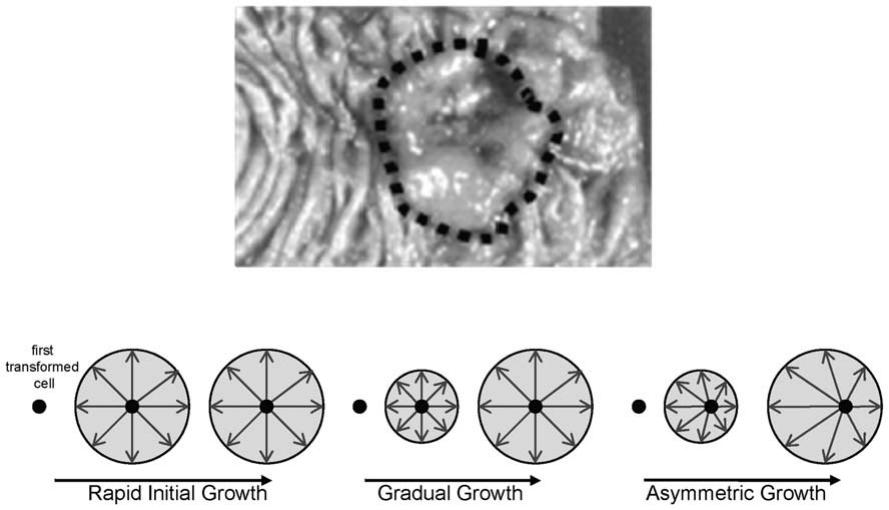
A picture of a colorectal adenocarcinoma with a cartoon illustrating three possible growth models: rapid growth, gradual growth, asymmetric growth.

Human cancers, such as colorectal cancers, are often relatively symmetric masses of tumor cells (Figure 1). They likely start from a single transformed cell, but how the progeny of this first cell eventually grow into a visible tumor is uncertain. One of the most fundamental characteristics of a human cancer, the number of cell divisions since transformation, or tumor ‘age’, is not directly measurable. For a long time we have understood that for solid tumors, a single transformed cell seeds the growth, and that present-day tumor cells are descendants from a clonal expansion of the first malignant cell [1]. Under such a growth model, the time to the most recent common ancestor of the cancer cell population is a measure of tumor age. This suggests the use of molecular phylogeny for inferring the tumor's past. Phylogenies are commonly used to estimate the time to the most recent common ancestor of related populations. Using molecular phylogeny to reconstruct the tumor's past will allow us to address basic questions about the unique histories of human cancers.

A common approach for developing phylogenies is the comparison of DNA sequence variation among different groups of organisms. However, within a human cancer, genetic variation due to somatic cell mutation is sufficiently rare that it is impractical to study. Instead, we focus on the epigenetic variation of DNA methylation [2]. DNA methylation is copied during somatic cell division and passed from generation to generation, with replication error (epigenetic ‘drift’). The differences in DNA methylation patterns that are introduced in the daughter cells record information on numbers of cell division. Since daughter cells are adjacent cells they should be the most similar in terms of epigenetic variation. At the same time, because a tumor is an expanding population, progeny move apart and cells on opposite halves of the tumor are likely only to be related near the initial growth phase in the distant past, so that their DNA methylation patterns should be the most distinct. Siegmund et al. (2009) [3] showed that by sampling cells from multiple locations in a colorectal tumor and combining information on epigenetic distance with physical distance, they could infer a tumor's ancestry.

**Figure 2:**
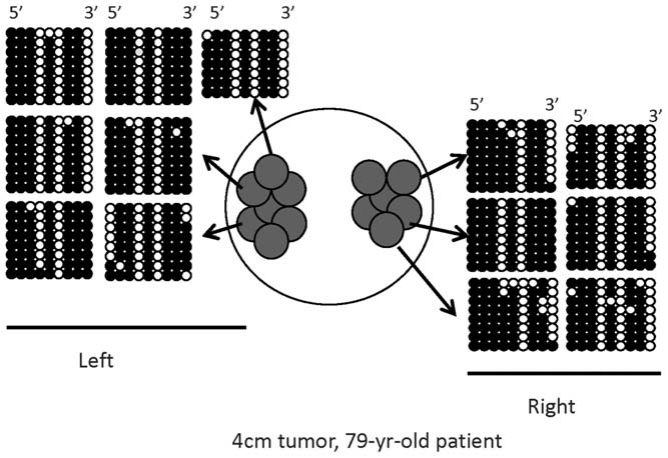
Tumor sampling scheme with bisulfite sequence data generated from one colorectal tumor. Grey circles denote cancer glands. Six to seven glands are sampled from each tumor half and multiple clones are sequenced for each tumor gland (e.g. 7–8). Black circles denote methylated CpGs and white circles unmethylated CpGs. Three physical distances: within a cancer gland, between cancer glands from the same tumor half, and between cancer glands from opposite tumor halves.

Physically, colorectal adenocarcinomas are comprised of glands that provide a natural substructure for (epi)genome sampling. By sampling multiple DNA sequences, from multiple glands in both the left and right side of the tumor, epigenetic distance can be computed between cells for three different physical distances: within a gland, between glands within a cancer half, and between glands from opposite cancer halves (see Figure 2). Using this approach, Siegmund et al. [3] observed smaller epigenetic distances within cancer glands than between glands, supporting the hypothesis that cells within a gland are more recently related than cells from different glands. They also found that for the majority of their colon cancers, epigenetic distances between glands are similar for glands sampled from opposite tumor halves and for glands sampled within the same tumor half, leading the authors to conclude that many cancers undergo a rapid initial, “homogenizing” expansion and that cancers are “flat” [3], [4]. However, in a subset of cancers where they observed smaller epigenetic distances within tumor half than between tumor half, they were unable to get a good fit of their growth model. Consequently, we are interested in evaluating different models for tumor growth that would allow for this alternate behavior. Specifically, we are interested in how variations in tumor physical growth may produce asymmetries in the epigenetic distances between tumor cells.

**Figure 3:**
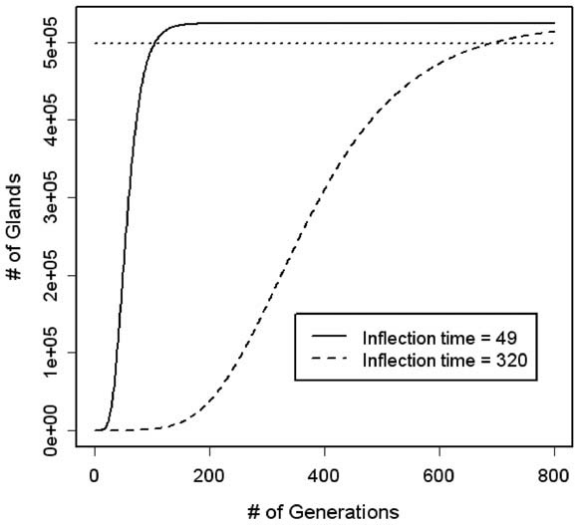
The number of glands in a tumor as a function of the number of generations of cell division for Gompertzian growth models with inflection times of 49 and 320 generations and a maximum of 524,288 glands.

Recent mathematical models for cancer growth focus on modeling single-cell behavior [5], [6], [7]. Our methodology is a model for cell ancestry that could be super-imposed on any single-cell model that simulates cell division. The tumor growth model we simulate is for a single clonal expansion, applying a constraint on the total number of cells. The models we consider are outlined in Figure 1. A tumor may grow uniformly, either quickly or slowly. Alternatively, there may be differences in division rates between tumor sides, such that one side has a different mitotic age. Tumor growth is modeled as a combination of two processes: one for cell division, and one for gland division [3]. An original transformed cell divides exponentially through a doubling process until it reaches sufficient numbers to populate a gland. As the cells continue to double in number, the glands also divide, so that the maximum number of cells allowed in a gland is not exceeded. We put a constraint on the total number of glands in the tumor, which we estimate based on the physical size of a tumor. Glands stop dividing when they reach this maximum number. In Siegmund et al. [3], the model for gland division is divided into two phases, one for exponential growth (during the cell doubling phase), and one for no growth. During this latter phase, the cells continue to divide within the glands, but cell death starts to occur allowing the total number of cells to remain constant. Although this two-phase model for gland division is a useful approximation for how a tumor might grow, a smooth growth trajectory is likely more realistic. In this paper, we model gland division using a Gompertzian, S-shaped growth curve (see Figure 3). A Gompertz growth curve is more flexible, allowing us to slow down the growth rate during the early phase of tumor development, and eliminate the discontinuity of a transition between exponential growth and no growth. This slowing down of the initial growth period might introduce the extra epigenetic variation seen in a subset of tumors. Alternatively, extra epigenetic variation may appear from asymmetric growth where tumor sides have different mitotic ages. Through a simulation study, we propose to assess the effect of different Gompertzian growth models, both symmetric and asymmetric, on the patterns of epigenetic tumor variation.

## Results

### Colon Cancer Data

Figure 2 shows data for one colon cancer. The measure of epigenetic distance increases with physical distance between the tags analyzed. The average Hamming distances are 0.62, 1.24 and 1.34, respectively, comparing tags within glands, tags within tumor halves (but between glands), and tags between tumor halves. This cancer is an example of a “flat” tumor, where we find similar distances between glands both within and between tumor halves. This suggests a rapid clonal expansion, such that all present-day glands are equally distantly related through the early, ancestral glands.

**Figure 4:**
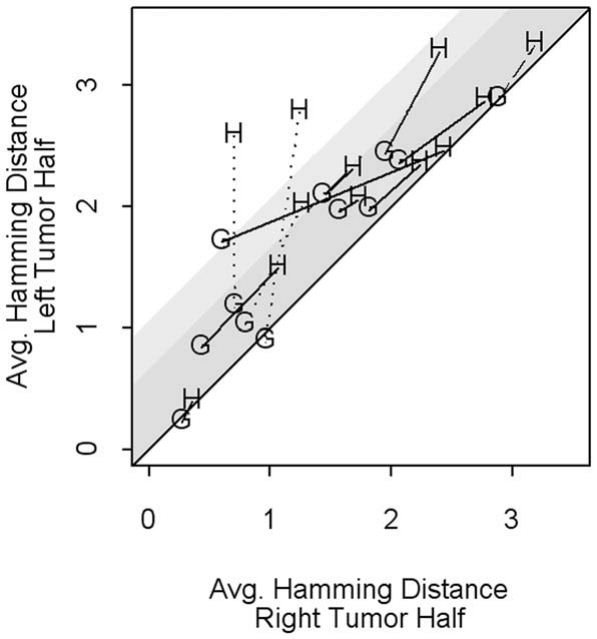
Average Hamming distance by tumor half for the 12 tumors published by Siegmund et al. [3] The x-axis represents the distance in the right tumor half and the y-axis the distance in the left tumor half. Within-gland distances are denoted by G and within-half by H. Lines connect the pairs (G,H) from the same tumor. The line type indicates differences in distance between-half and within-half (BH-WH). Solid lines denote small BH-WH differences (difference <0.1, 8 “flat” tumors) and broken lines denote larger differences (BH-WH difference >0.4, 4 tumors). The type of broken line indicates WH differences between left and right tumor half; dotted lines indicate tumors with large WH differences (>0.6, 3 tumors); the dashed line indicates one tumor with small WH difference (0.14).

Figure 4 displays the average Hamming distances by tumor half for all twelve tumors. The within-gland distances for a tumor are denoted by G and the within-half distances by H. Lines connect the distances from the same tumor. For all tumors, the average within-gland distance is smaller than the average within-half distance, as reported by Siegmund et al. [3], suggesting that cells within a gland are more closely related than cells from different glands. Lines that lie close to the diagonal show within-gland distances and within-half distances that are similar in the left and right halves. This pattern is expected from tumors that underwent a similar numbers of cell division in each tumor half (“symmetric growth”). Lines that fall off of the diagonal show the average Hamming distance is higher in one tumor half. (We arbitrarily assign the larger distance to the left half.) This pattern suggests that the tumors grew asymmetrically and that one half underwent more cell division than the other. The eight “flat” colorectal tumors with a between-half distance similar to a within-half distance are denoted by solid lines. The remaining four are denoted by broken lines. Because of the correlation structure of the data (all cells descend from a common ancestor), we are unable to assign statistical significance to a test for symmetric growth. Therefore we investigate, through simulation, various models that will allow us to observe different patterns of epigenetic variation, and characterize how variable those patterns can be. We begin with a thorough exploration of models undergoing symmetric growth, exploring the effect of the rate of tumor growth (slow versus fast), and consider at the end the variation in patterns that can be induced if growth is asymmetric.

### Symmetric Growth

#### Fraction of tumor sampled

**Figure 5:**
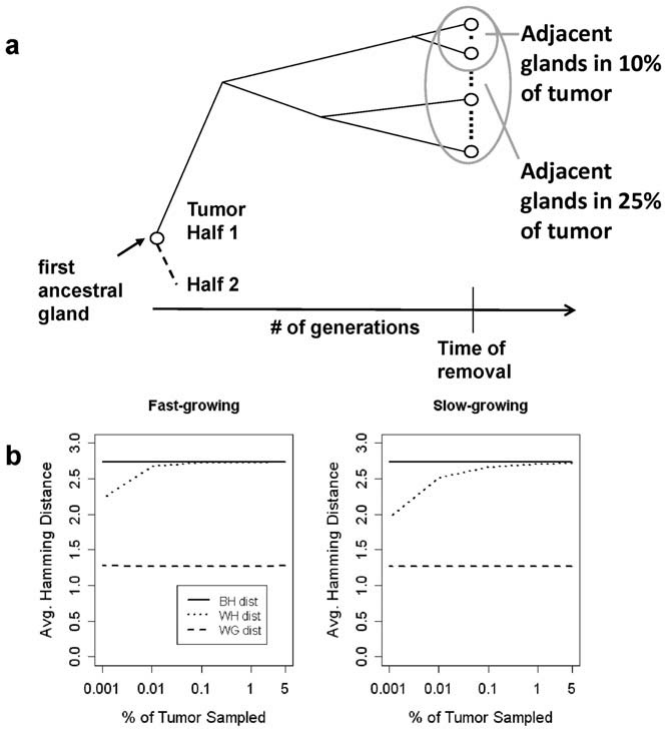
The relationship between physical proximity in the tumor and epigenetic distance. (a) The relationship between the fraction of tumor sampled and time to the most recent common ancestor. (b) Mean estimates of average Hamming distance for three physical distances: between tumor half (BH), within tumor half (WH) and within gland (WG) under different growth curve inflection times (inflection time is 49 for figure on left and 320 for figure on right) (N = 10,000 replicates).

First, we evaluate whether the sizes of the tumor samples affect our measure of epigenetic distance, the average Hamming distance. Figure 5a shows a schematic that illustrates our model for sampling glands from tumor halves. By restricting the size of the cancer fragment from which we sample the glands, we obtain some control over the time to the most recent common ancestor of the sampled glands. The smaller the size of the initial cancer fragment, the more recent is the time at which the sampled glands coalesce to a common ancestor. And the more recent the common ancestor, the smaller the epigenetic distance between glands becomes (Figure 5b). In order to mimic sampling from opposite tumor halves, we sample from the top-most and the bottom-most branches of the tree. Figure 5b shows the three mean estimates of the average Hamming distance from a simulation study, when sampling glands from different fractions of the tumor in each half. First, we see that the mean estimates of average Hamming distance increase with physical distance in the tumor (within-gland<within-half<between-half). Neither the within-gland distances nor the between-half distances are sensitive to the size of the cancer fragment from which we draw our samples, but the within-half distance is. The distance decreases the smaller the fraction of tumor sampled. The within-half distance is also sensitive to the rate of tumor growth. The slowing down of tumor growth has the intended effect of decreasing within-half distance compared to between-half distance. This is most apparent when studying small cancer fragments.

In our study, the two experimentally sampled cancer fragments represent 5% or more of the total tumor volume. Figure 5b shows little difference in within-half and between-half distances for cancer fragments of this size. Nevertheless, we sample from the extreme 5% of the tumor for the remainder of our results section (2.5% per half) in order to maximize our chances of seeing smaller within-half than between-half distances for slow-growing tumors.

#### Epigenetic distances under different growth models

**Figure 6:**
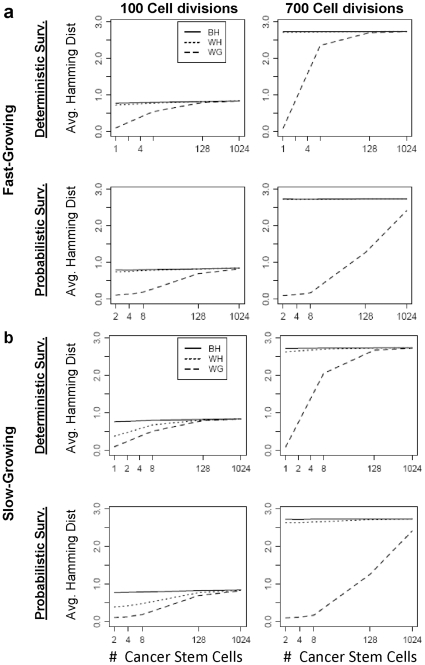
Mean estimate of average Hamming distance between-half (BH), within-half (WH), and within gland (WG) from a simulation study (N = 10,000 replicates). Growth curve inflection time is 49 generations of cell division for fast growing tumors and 320 generations for slow-growing tumors.

We simulate data for tumors having different growth rates, models for cell division, and numbers of cancer stem cells (CSCs) at various stages of their development (numbers of generations of cell division) and plot the average estimates for the three different epigenetic distances: between half (BH), within half (WH) and within gland (WG) for various parameter settings (Figure 6). Focusing first on the estimate of BH distance and comparing results in the right column to results in the left column, we see that the BH distance is higher at 700 generations of cell division than at 100 generations. In fact we see a general trend of increasing BH distance with increasing number of cell divisions through 1000 generations (results not shown). Looking within a column at models for tumors of the same age, the average BH distance does not vary by number of CSCs (slope = 0) or the type of cell division and tumor growth rate (constant intercept). These suggest that for a symmetric tumor and known DNA methylation error rates, the tumor's age is estimable using the information on between-half distance alone.

In contrast, the estimate of within-gland distance is a function of multiple parameters. WG distance increases with tumor age when there are multiple CSCs seeding a cancer gland. This behavior reflects the epigenetic drift occurring to the CSCs. If there is a single CSC seeding a gland, all non-CSCs are closely related resulting in little epigenetic variation between epialleles within a gland. Drift in the DNA methylation pattern of the long-lived CSC has a negligible effect on the within-gland distance. However, if a gland has two (or more) long-lived CSCs that drift apart from one another over time, the descendants of each will have epigenetic patterns similar to their ancestral cell, and will show differences from the descendants of the other CSC. On average, the number of differences increases with the number of divisions separating the CSCs, resulting in an increased WG distance with increased tumor age. Siegmund et al. [3] reported this behavior for their data, supporting a model for multiple CSCs.

For a tumor of a given age, two parameters influence the within-gland distance: the number of CSCs and the type of cell division (deterministic or probabilistic). These two parameters are not identifiable from the WG distance alone. Compared to growth under a deterministic model for cell division, a probabilistic model with a higher numbers of CSCs will yield similar WG distance estimates. The growth rate is also seen to play a role, but only under the deterministic model for cell division; under the deterministic model, higher WG distances are seen under faster growing tumors. Under a model for probabilistic cell division, the WG distance appears to be insensitive to the tumor growth rate.

Lastly, we report on the within-half distance, and for which scenarios it is most different from between-half distance. The largest differences in mean estimates of the WH and BH distances occur for slow growing tumors that are both young (have not achieved their maximum size), and have only a few to moderate number of CSCs. If the majority of cells are CSCs, there is a lot of diversity even within a tumor half such that the within-half distances are just as large as the between-half distances. Also, as the tumors age, the WH distance approaches the BH distance, and by the time the tumor is full grown, even for the slow-growing tumor with few CSCs, the two distances are approximately equal for our sampling design. This suggests that a slower Gompertzian growth rate cannot account for the larger BH-WH distances that we observe in four of our tumors (differences = 0.47, 0.68, 0.80, 1.63), and motivates us to explore whether asymmetric growth, where one tumor half undergoes more generations of cell division than the other, could explain such differences.

### Asymmetric growth

Under asymmetric growth, we expect the within-half distance to differ in the two tumor halves, and the between-half distance to be greater than the average within-half distance, and more similar to the within-half distance from the faster-growing half. We investigate this possibility by simulating tumors which undergo different numbers of cell division in their two halves. First, we determine if larger BH than WH distances can arise under asymmetric growth. Second, we evaluate the variability in WH distances between left and right tumor half, under both symmetric and asymmetric growth models. For the latter scenario, we simulate data for 1,000s of tumors under a symmetric growth model and compute the (absolute) difference in WH distance between left and right tumor half to assess normal variation. The 95^th^ percentile from this distribution is used to infer asymmetric growth for a tumor with an observed difference that exceeds this value. The power of the test is assessed from simulations of tumors that grow asymmetrically.

#### BH-WH distances

We simulate 100, 400, and 700 generations of cell division in each tumor half under the probabilistic survival model, fixing the number of CSCs per gland at 128 and 1024. One thousand replicate data sets are generated for each scenario.

On average, the difference between the between-half and the within-half distances is greater under asymmetric growth than under symmetric growth. For this we compare tumors that have undergone the same total number of cell divisions in both halves. e.g. 100 and 700 generations in the left and right half, respectively, compared to 400 generations in each half, for a total in each tumor of 800 generations. The median BH-WH difference for the tumors under asymmetric growth is 0.45 (95% simulation interval (SI) = 0.09–1.0) under 128 CSCs and 0.44 (95% SI = 0.21–0.80) under 1024 CSCs. These are greater than the differences under symmetric growth (see Figure 6). Furthermore, these differences are large enough to explain the four “non-flat” tumors. Next we investigate the variation in within-half distances between left and right tumor halves.

#### Left – right WH distances

We assess asymmetric growth by comparing the absolute difference in WH distance in the left and right tumor half to the upper bound (95^th^ percentile) of the distribution under a symmetric growth model. Under symmetric tumor growth, the absolute difference of the WH distance increases with increasing numbers of generations. For 1024 CSCs, the 95^th^ percentiles of the absolute differences are 0.552, 0.606, and 0.613 for 100, 400, and 700 generations, respectively. This suggests that an absolute difference in Hamming distance greater than about 0.6 is needed to suggest asymmetric growth. As expected, the degree of asymmetry of tumor growth dictates how frequently we see such variation. When one tumor half undergoes 100 generations and the second undergoes 700 generations, all 1,000 simulated tumors had an average (absolute) difference in WH distance that exceeded 0.6. However, if the tumor halves are more close in age (400 and 700 divisions), this much variation is relatively rare (19%). If there are fewer CSCs, more variation between the two halves is observed (left-right WH difference = 0.863, 0.987, and 1.018, respectively). Under this model, tumors that show a WH difference greater than 1.0 would show evidence for asymmetric growth.

Two tumors show variation between tumor halves exceeding the more stringent criteria of 1.0 for concluding asymmetric growth (Figure 4). These are the points labeled as H appearing in the upper left triangle of Figure 4, where the difference between the y-axis and x-axis coordinates exceeds one. Using the less stringent criteria of 0.6, five tumors show variation between tumor halves exceeding random variation (WH difference >0.6, points H appearing in both the white and light grey area). Three of these five tumors also show large BH-WH differences (>0.4) (Figure 4, dotted lines). Although two tumors do not, this is not surprising given the wide 95% simulation intervals reported earlier under asymmetric growth models and suggests a lack of power to detect asymmetric growth. Together, these results suggest that as few as two, but as many as five of the tumors may show evidence of asymmetric growth.

## Discussion

Reconstructing tumor histories from epigenomic variation may be difficult if outcomes are highly sensitive to small variations in tumor growth, or if underlying ancestries are complex. For example, methylation patterns may be extremely complex if primary CRCs grow through sequential clonal evolution because each clonal expansion would have a different mitotic age and diversity. However, experimental data from human CRCs reveal that passenger DNA methylation pattern variation from different parts of the same tumor are often similar [3], suggesting underlying ancestries may be limited to relative symmetric growth patterns (i.e. single clonal expansions). Biologically, it is unlikely that tumors will grow perfectly symmetrical as some parts of the same tumor are likely to grow faster than others due to different microenvironments. We investigated this potential problem by modeling how different tumor growth rates effect methylation pattern variation. The model for tumor growth allows cancer glands to divide following a smooth, Gompertzian growth curve. Under a Gompertzian model, the rate of tumor growth is parameterized by the time of the maximum growth rate (inflection time). We consider two extremes of this model. On the one extreme we have a fast-growing tumor where the initial growth is constrained such that it will not grow faster than a doubling of cancer glands at each generation (exponential growth). On the opposite extreme, we pick a more slow-growing tumor, where the tumor grows to within 5% of its maximum size by a fixed number of generations. In order to maximize our chances for finding differences in epigenetic distances within a tumor half and between tumor halves, we sampled from the extreme 5% of our cancer tree, the smallest cancer fragment size that is likely to represent real tumor fragments.

In simulations, we find that regardless of the rate of tumor growth, for tumors that have achieved their maximum size, at least approximately, WH distance≈BH distance. This is consistent with what we observe in eight of our tumors. In another three tumors, differences between WH and BH distance may be attributed to the asymmetric growth of the two tumor halves, albeit sequential clonal evolution could also explain how passenger methylation patterns may differ between tumor halves. Then the data from only one tumor cannot be explained by our model. These simulation studies help explain why experimentally obtained DNA methylation pattern data typically reconstruct symmetrical tumor growth, because epigenomic variation is relatively insensitive to growth rates or regional differences in growth rates. Although the simulations illustrate that detailed information on tumor growth will be difficult to infer from tumor methylation patterns, this drawback should be tempered by current absence of methods to infer human tumor histories.

The inability to model all tumors is evidence for further dependencies in our data that are not captured by our model, including experimental problems with adequately sampling epialleles from human tumors. Such dependencies would allow for greater variation in the data we observed, perhaps permitting us to see larger differences between WH and BH distances. As a next step, we propose to relax the assumption that all CpG sites are methylated independently of one another. In fact, one study has found that DNA methylation errors are more likely to be added to tags that have a lot of DNA methylation present [8]. Unfortunately the data in this study is not sufficient to explore different dependence structures. A similar conclusion was reached in a second study [9], however their analysis did not take into account the mixture of alleles that arises when studying autosomal loci. We are in the process of obtaining new bisulfite sequence data from cell lines which will allow us to investigate different models in detail. Our future work will be to focus on better understanding, and modeling of the DNA methylation copy errors.

Several investigators have proposed single-cell models for tumor growth [6], [7], [10]. Like their models, our model mimics actual cell division. Unlike their models, ours provides estimates of tumor age and number of dividing cells for individual tumors. These estimates are obtained using approximate Bayesian computation, a simulation-based approach [2], [11]. Our data are inconsistent with a recent report suggesting that tumors are conglomerates of self-metastases [7]. Such a sequential growth pattern would likely generate greater heterogeneity in epigenomic variation than we observe in our data, with older tissue sections showing greater diversity than more recent conglomerates. Instead, we find that the majority of our tumors appear “flat”, with similar measures of diversity in the outermost left and outermost right tumor sides. Such behavior can be explained by a single clonal expansion.

In our model there are six parameters, two of which are the probabilities that DNA methylation copy errors occur at a given CpG site (methylation and demethylation errors). In these types of models, the estimates of DNA methylation copy errors and the total number of generations of cell division are not identifiable. For a given set of data (and parameters), similar data can be generated after a doubling of the copy error rates and halving of the number of generations. Siegmund et al. [3] suggested specifying an average tumor from a set of tumors, and calibrating the error estimates to agree with that average. For the sake of focusing this paper, we do not address this issue further here. Instead, we fix the DNA methylation copy error rates at plausible values for the *BGN* locus, and evaluate the effect of other parameters on our data. In practice, we will estimate the copy error rates from the data by a proper calibration.

Other simplifications we have made to the model is to assume non-overlapping generations. Inclusions of overlapping generations is conceptually straightforward and will be explored in future work.

In conclusion, we present a biologically plausible model that allows us to explore the unobserved ancestries of human CRCs. Experimental data constrains our modeling such that a single clonal expansion after transformation is usually sufficient to explain the methylation pattern diversities observed in different parts of the same primary CRC. Further studies with more data may better reconstruct exactly how individual human tumors grow.

## Materials and Methods

### Colon cancer data

In Siegmund et al. (2009), bisulfite sequence data was generated for two X chromosomal regions in tumors from 12 male patients. Restricting the study to male patients simplifies the statistical analysis because in males, cells only carry one copy of the X chromosome. Therefore, the cell that underwent the initial transformation to cancer carried a single X chromosome and all X chromosomes in the cancer are descendants from that original copy. Bisulfite sequence data were obtained from sampling six or seven glands each from opposite tumor halves (see Figure 2). The glands are sampled from cancer fragments that are a minimum of 2 cm apart. Cancer glands contain ∼2,000–10,000 cells, and eight clones are sequenced from each gland. In this paper we focus on data for 9 *CpG* sites sequenced in the *Byglycan* (*BGN*) region. The data for a single site are coded 0–1, where “1” denotes methylated and “0” unmethylated. A ‘tag’ refers to the code for a string of *CpG* sites. An unmethylated tag is represented by “000000000”. When studying n *CpG*s, there are a total of 2^n^ possible tags.

This study was approved by the Institutional Review Board of the University of Southern California Keck School of Medicine. Written informed consent was obtained from all study participants.

### Data simulation

#### Growth model

**Figure 7:**
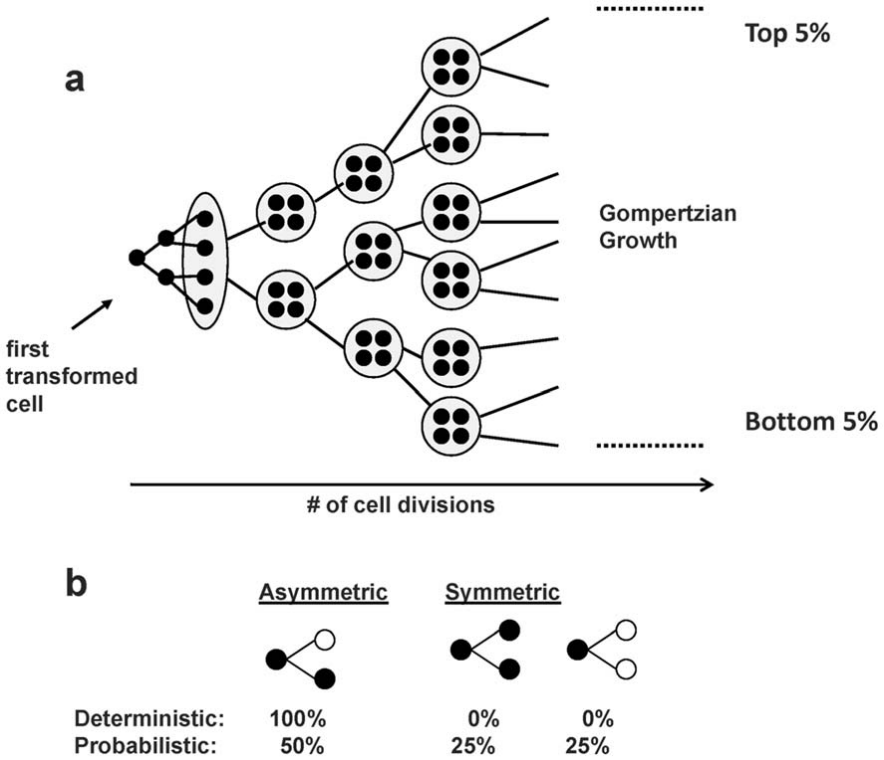
Schematic of sample genealogy. (a) Example genealogy. Black circles denote cancer stem cells and grey circles denote cancer glands. (b) Two models for cell division. Black circles denote CSCs and white circles denote non-CSCs.

Tumor growth is modeled by two branching processes. We specify a model for cell division that is nested within a model for tumor glands. For both processes, we assume, for convenience, that the generations are discrete; however, this assumption can be relaxed and overlapping generations permitted. The entire process follows a tree topology similar to the one shown in Figure 7a. In Figure 7a, time runs from left to right across the page, with the left-most cell being the original transformed cell from which the others grow. In an initial growth phase, the original transformed cell undergoes a period of exponential growth (cell doubling) until it forms a small population that comprises a cancer gland. In subsequent generations, the two growth processes (cellular and glandular) occur simultaneously. The number of cancer glands follows a Gompertzian growth model, a smooth S-shaped curve. At generation k, the number of glands, N_k_, is given by
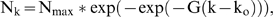
where N_max_ is the maximum number of glands and is estimated based on tumor size, G is the growth rate, and k_o_ is the inflection time point, or the time of maximum growth. For any inflection time point k_o_, we can estimate the growth rate G, by substituting N_k_ = 1 at generation k = 1. Earlier research suggests that there are approximately one billion (10^9^) cells per cm^3^ of tumor and 8,192 cells per gland. To simulate tumors that are approximately 4 cm^3^ in size, we set as a maximum 524,288 glands (524,288 glands×8,192 cells/gland ∼4.3×10^9^ cells). Figure 3 shows examples of growth curves that could generate, after 700 generations, a tumor with ∼½ million glands. The possible Gompertzian growth models are restricted so that the growth rate never results in more than a doubling of the number of cancer glands in any single generation. Therefore, a fast-growing tumor will approximately double in size at each generation during its early development, before slowing down and eventually reaching a plateau at some maximum size (N_max_) (solid line Figure 3). A slow-growing tumor will start off slowly, never doubling in size at any single generation, but growing along a smooth S-shaped curve, until attaining the fixed maximum size. In Figure 3, the inflection time for the slow-growing tumor (dashed line) is chosen so that the tumor will be within 5% of its maximum size at 700 generations.

During the Gompertzian growth phase, individual cells acquire different behavior. Some continue to divide with unlimited proliferative potential while others divide, but for only a fixed number of generations. We refer to the former cell type as cancer stem cells (CSCs) and the latter as non-cancer stem cells. Allowing for cells with different behavior, allows the cells to continue ‘aging’ in a tumor of a fixed size. We fix the number of CSCs and non-CSCs to constants for all glands in a tumor. These represent the average number of CSCs and non-CSCs, taken across all glands. One possible extension of our model would include the variability in these numbers across glands to capture random fluctuations.

At each generation cells divide, however the manner in which they divide will depend on whether the gland divides, or simply ‘ages’. If a gland divides, the cells divide producing two daughter cells of the same type (e.g. a CSC produces two CSCs and a non-CSC produces two non-CSCs). The number of cells in the gland is doubled, and the offspring segregate at random as the gland divides into two offspring glands. If the gland does not divide, the cells follow a division process described in Figure 7b. The two models considered allow the cells within a gland to continue to ‘age’, without growing in number. In the deterministic model CSCs divide asymmetrically, always leaving one CSC and one non-CSC as daughter cells. In the probabilistic model, CSCs divide either asymmetrically or symmetrically, each with 50% probability. Symmetric divisions of a CSC into two CSCs or into two non-CSCs are both given equal probability (25% each). This probabilistic model for cell division has been used previously to study stem cell behavior in normal colonic crypts [12].

Overall, there are six parameters in our model: (i) the inflection time for the Gompertzian growth model, (ii) the total number of generations of cell division, (iii) the number of CSCs per gland (constraining the total number of cells in a gland to 8,192), (iv) the type of cell division within a non-dividing gland (100% asymmetric or 50%), and (v–vi) two DNA methylation copy error rates (methylation and demethylation error rates). The details of the simulation process and the modeling of DNA methylation copy errors are described next.

#### Simulating the branching process

The simplest way to simulate tumor growth is to model the entire branching process and then randomly sample cells from the glands sampled in the final generation. However, because a human tumor of moderate size contains ∼4.3 billion cells, this will surpass memory limits on standard desktop computers. Consequently, we have adopted a more efficient, genealogical perspective in which we model only the ancestry of the sampled cells (drawn from the rightmost generation in Figure 7a). First, we generate the shape of the ancestry for the glands sampled from our final generation (e.g. 14 glands); then we simulate the evolution of the cell populations along that ancestry. The details follow:

1. For each generation k, we estimate the number of glands N_k_ from the Gompertz equation, storing the entire ancestry for all glands. Although the order of branches for the division of any single gland is symmetric, the glands from the top of the tree will be more similar to one another than they will to glands from the bottom of the tree, and vice versa, since glands from the top and bottom are only related through their shared ancestry back to the original ancestral gland. Thus, by considering the top of the tree as one half of the tumor and the bottom of the tree as the other half, we have created a local similarity of glands from the same tumor half. To mimic the sampling of glands from opposite halves of the tumor, we restrict our sampling in the final generation to glands from the top-most and bottom-most branches of the tree (e.g. In Figure 7a, the top and bottom 2.5% of glands). Then we save the ancestry for only the sampled glands.
2. Next we model the evolution of cells along the ancestry of our sampled glands. First, we notice that we do not need to model all cells in all generations because the CSCs are the only long-lived cells in our tree. Since each gland must have the pre-defined number of CSCs out of the total 8192 cells, the number of non-CSCs can never be greater than 8192 less that number. Furthermore, since a gland can never exceed the fixed total number of cells, non-CSCs can never undergo more than 12 generations of division (see Table 1). Therefore we do not need to generate non-CSCs in the early generations of our ancestry, because they will not contribute to the population alive at the end of tumor growth. We only generate non-CSCs in the final n generations, where n depends on the number of CSCs (see Table 1). For the early generations, we only need to simulate data for the CSCs.
  We begin by simulating the evolution of the first transformed cell into the first cancer gland. Here we are modeling a simple branching process for a haploid genome, whereby at each generation *k* the cell copies itself from generation *k−1*, with possible changes in methylation status at each locus (We discuss the details for the starting methylation pattern and the way in which methylation status changes below.) The first gland is formed when there are enough CSCs cells to populate it. In each subsequent generation of growth, cells divide in a manner that keeps the total number of CSCs (and non-CSCs) per gland constant. As described earlier, if a gland divides, all cells divide producing two daughter cells of the same type and the offspring segregate at random as the gland divides into two offspring glands. If the gland does not divide, the cells follow a division process parameterized by r, the probability of an asymmetric division as described in Figure 7b. We consider two particular models having biological appeal: (1) deterministic (immortal) cancer cell survival (r = 1) and (2) probabilistic (random) cancer cell survival (r = 0.5). Under the probabilistic model, a CSC has a 25% chance of dividing into two CSCs and a 25% chance of dividing into two non-CSCs, with a constraint of having a constant number of CSCs in each generation. The constraint is implemented by computing the probabilities of possible patterns for cell division for any fixed number of CSCs. These joint probabilities are determined by a convolution formula. At each generation the CSCs are selected by sampling without replacement, conditional probabilities are assigned, and the cell's behavior is determined. For example, if the required number of CSCs for the next generation is reached, the remaining CSCs are forced to yield non-CSC offspring only. For complete details on the approach, see the supplemental information from [13].

**Table 1:** The maximum number of generations that non-CSCs divide when there is a maximum of 8,192 cells per gland.

No. of CSCs	No. of Generations Non-CSC divides
4096	0
2048	1
1024	2
512	3
256	4
128	5
64	6
32	7
16	8
8	9
4	10
2	11
1	12

For cancer, the DNA methylation pattern of the original transformed cell is unknown. As our goal is to model tumor growth from copy errors that accumulate with cell division, and because most cancers occur in older individuals, the original transformed cell is likely to have accumulated some baseline DNA methylation levels. We simulate data for a single *CpG* region that includes 9 *CpG*s, assuming that 6 of the 9 measured *CpG*s are methylated in the original transformed cell (∼67%). We use this because it represents the average global percent methylation seen in the majority of our tumors. In future work, we will consider simulating data over a variety of starting values to understand their effect on the joint behavior of the summary statistics.

We model DNA methylation copy errors at each *CpG* independently according to some constant probabilities. We parameterize these with (μ,δ), where μ denotes the probability with which an unmethylated *CpG* becomes methylated and δ the probability with which a methylated *CpG* becomes unmethylated. For these simulations, we fix these parameters at 0.001 and 0.00025, values that seem reasonable based on earlier analyses of one of the X chromosomal regions (*BGN*). We expect that for different regions of the genome the error rates will differ, and extending these methods to multiple loci is an important direction for future work. We focus in this paper, on the effects on our data of the four remaining model parameters: (i) the inflection time point for the Gompertzian growth curve (whether the tumor is fast or slow growing), (ii) total number of cell divisions (tumor ‘age’), (iii) the number of CSCs per gland, and (iv) the type of cell division (deterministic or probabilistic).

### Summary statistics

For any large data set, stochastic variation will make it impractical to simulate data that replicate the exact DNA methylation patterns we observe. Therefore, we assess our ability to simulate data that mimic real data by a comparison of summary statistics. Siegmund et al. found the average Hamming distance to be the most informative parameter of tumor diversity [3]. Hamming distance is defined as the number of sites that differ between two tags. For example, the distance between 0011 and 0110 is two. In a simulation study, we assess the impact of different growth model parameters by their effect on the average Hamming distance between tags sampled at three different physical distances: within gland, between glands but within half, and between half.
